# Analysing diversification dynamics using barcoding data: The case of an obligate mycorrhizal symbiont

**DOI:** 10.1111/mec.16478

**Published:** 2022-05-05

**Authors:** Benoît Perez‐Lamarque, Maarja Öpik, Odile Maliet, Ana C. Afonso Silva, Marc‐André Selosse, Florent Martos, Hélène Morlon

**Affiliations:** ^1^ Institut de biologie de l’École normale supérieure (IBENS) École Normale Supérieure CNRS INSERM Université PSL Paris France; ^2^ Institut de Systématique, Évolution, Biodiversité (ISYEB) Muséum National d’histoire Naturelle CNRS Sorbonne Université EPHE, UA, CP39 Paris France; ^3^ University of Tartu Tartu Estonia; ^4^ University of Lille CNRS, UMR 8198 ‐ Evo‐Eco‐Paleo Lille France; ^5^ Department of Plant Taxonomy and Nature Conservation University of Gdansk Gdansk Poland

**Keywords:** arbuscular mycorrhiza, ecological niche, fungi, macroevolution, microbial diversification, obligate symbiosis

## Abstract

Analysing diversification dynamics is key to understanding the past evolutionary history of clades that led to present‐day biodiversity patterns. While such analyses are widespread in well‐characterized groups of species, they are much more challenging in groups for which diversity is mostly known through molecular techniques. Here, we use the largest global database on the small subunit (SSU) rRNA gene of Glomeromycotina, a subphylum of microscopic arbuscular mycorrhizal fungi that provide mineral nutrients to most land plants by forming one of the oldest terrestrial symbioses, to analyse the diversification dynamics of this clade in the past 500 million years. We perform a range of sensitivity analyses and simulations to control for potential biases linked to the nature of the data. We find that Glomeromycotina tend to have low speciation rates compared to other eukaryotes. After a peak of speciations between 200 and 100 million years ago, they experienced an important decline in speciation rates toward the present. Such a decline could be at least partially related to a shrinking of their mycorrhizal niches and to their limited ability to colonize new niches. Our analyses identify patterns of diversification in a group of obligate symbionts of major ecological and evolutionary importance and illustrate that short molecular markers combined with intensive sensitivity analyses can be useful for studying diversification dynamics in microbial groups.

## 1 INTRODUCTION

Understanding past dynamics of speciation and extinction, as well as the abiotic and biotic factors that modulate the frequency of speciation and extinction events (i.e., diversification rates), is key to understanding the historical processes that shaped present‐day biodiversity patterns (Barnosky, 2001; Benton, 2009; Chomicki et al., 2020; Clarke & Gaston, 2006; Condamine et al., 2018; Morlon, 2014; Varga et al., 2019). While phylogenetic analyses of diversification are widespread in well‐characterized groups of species, such as animals and plants (Givnish et al., 2015; Magallón & Sanderson, 2001; Rolland et al., 2014; Upham et al., 2019), they are much more challenging in groups for which diversity is mostly known through environmental DNA sequences and molecular techniques. In particular, the characterization of poorly cultivable microbial groups such as most bacteria and fungi is often limited to metabarcoding techniques, which consist of specific amplification and sequencing of a short DNA region (Taberlet et al., 2018). On the one hand, these data often make species delineation, phylogenetic reconstruction and the estimation of global‐scale diversity highly uncertain, which all affect the phylogenetic inference of diversification dynamics (Lekberg et al., 2018; Moen & Morlon, 2014). On the other hand, it is possible to assess the robustness of phylogenetic diversification analyses to data uncertainty. Given the current limitations of sequencing technologies and the nature of the molecular data available for most microbial groups, using metabarcoding data and performing thorough robustness analyses is one of the only (if not the only) possible approach to analyse their diversification dynamics (Davison et al., 2015; Lewitus et al., 2018; Louca et al., 2018).

Here we analyse the diversification dynamics of arbuscular mycorrhizal fungi from the subphylum Glomeromycotina. These fungi are obligate symbionts that have been referred to as an “evolutionary cul‐de‐sac, albeit an enormously successful one” (Malloch, 1987; Morton, 1990). This alludes to their ecological success despite limited morphological and species diversities: they associate with the roots of >80% of land plants, where they provide mineral resources in exchange for photosynthates (Smith & Read, 2008). Present in most terrestrial ecosystems, Glomeromycotina play key roles in plant protection, nutrient cycling and ecosystem processes (van der Heijden et al., 2015). Fossil evidence and molecular phylogenies suggest that Glomeromycotina contributed to the emergence of land plants (Feijen et al., 2018; Field et al., 2015; Selosse & Le Tacon, 1998; Strullu‐Derrien et al., 2018) and co‐evolved with them for more than 400 million years (Myr) (Lutzoni et al., 2018; Simon et al., 1993; Strullu‐Derrien et al., 2018).

Glomeromycotina are microscopic soil‐ and root‐dwelling fungi that are hard to differentiate based on morphology and difficult to cultivate without the host plant. Although their classical taxonomy is based mostly on the characters of spores and root colonization (Smith & Read, 2008; Stürmer, 2012), Glomeromycotina species delineation has greatly benefited from DNA sequencing (Krüger et al., 2012). Experts have defined “virtual taxa” (VT) based on a minimal 97% similarity of a region of the 18S small subunit (SSU) rRNA gene and monophyly criteria (Öpik et al., 2010, 2014). As for many other pragmatic species delineation criteria, VT have rarely been tested for their biological relevance (Powell et al., 2011), and a consensual system of Glomeromycotina classification is still lacking (Bruns et al., 2018). Besides the rDNA region, Glomeromycotina remain poorly known genetically: other gene sequences are available for only a few species (James et al., 2006; Lutzoni et al., 2018) and fewer than 30 complete genomes are currently available (Venice et al., 2020).

Hence, despite the ecological ubiquity and evolutionary importance of Glomeromycotina, large‐scale patterns of their diversification dynamics, as well as the factors that correlate with these dynamics, remain poorly known. A previous dated phylogenetic tree of VT found that many speciation events occurred after the last major continental reconfiguration around 100 million years ago (Ma) (Davison et al., 2015), suggesting the radiation of Glomeromycotina is not linked to vicariant speciation during this geological event. Indeed, vicariant speciation might play only a minor role in Glomeromycotina diversification, as these organisms have spores that disperse efficiently, promoting gene flow (Bueno & Moora, 2019; Correia et al., 2019; Egan et al., 2014). Based on the diversity and abundance of Glomeromycotina in tropical grasslands (Read, 1991), it has been suggested (but never tested) that these habitats are diversification hotspots for Glomeromycotina (Pärtel et al., 2017). In this case, the pace of Glomeromycotina diversification through time could be tightly linked to changes in the total area of tropical grasslands. Finally, Glomeromycotina are currently obligate symbionts and their evolutionary history could thus have been largely influenced by their interactions with their host plants (Lutzoni et al., 2018; Sauquet & Magallón, 2018; Zanne et al., 2014). Over the last 400 Myr, land plants have experienced massive extinctions and radiations (Cleal & Cascales‐Miñana, 2014; Zanne et al., 2014), adaptations to various ecosystems (Bredenkamp et al., 2002; Brundrett & Tedersoo, 2018), and associations with different soil microorganisms (Werner et al., 2014, 2018). All these events could have influenced the diversification dynamics of Glomeromycotina, although their relative generalism (van der Heijden et al., 2015; Perez‐Lamarque et al., 2020; Sanders, 2003) could buffer this influence.

Here we aim to characterize the pace of Glomeromycotina diversification in the last 500 Myr and to test the association between diversification rates and a variety of biotic and abiotic factors. We begin by reconstructing several thoroughly sampled phylogenetic trees of Glomeromycotina, considering several criteria of species delineations and uncertainty in phylogenetic reconstructions. We combine this phylogenetic data with palaeoenvironmental data and data regarding current Glomeromycotina geographical distributions, ecological traits, interactions with host plants and genetic diversity. Finally, we apply a series of birth–death models of cladogenesis to answer specific questions and test hypotheses related to Glomeromycotina diversification: (i) How often do speciation events occur? (ii) Were speciation rates relatively constant, or were they higher during specific periods of evolutionary history, and do speciation rates decline through time, as observed for many macroorganisms (Moen & Morlon, 2014)? (iii) Are speciation rates positively correlated with past temperature, CO_2_ concentration and/or land plant diversity? (iv) Are present‐day speciation rates correlated with geographical distribution, spore size (itself often inversely related to dispersal capacity, Nathan et al., 2008), degree of specialization toward plant species and genetic diversity? For each of these questions, we thoroughly assess the robustness of our results to uncertainty in the data.

## 2 MATERIAL AND METHODS

### 2.1 Virtual taxa phylogenetic reconstruction

We downloaded the Glomeromycotina SSU rRNA gene sequences from MaarjAM, the largest global database of Glomeromycotina gene sequences, updated in June 2019 (Öpik et al., 2010). We reconstructed several Bayesian phylogenetic trees of the 384 VT from the corresponding representative sequences available in the MaarjAM database (Methods S1). We used the full length (1700 bp) SSU rRNA gene sequences from Rimington et al. (2018) to better align the VT sequences using mafft (Katoh & Standley, 2013). We selected the 520‐bp central variable region of the VT aligned sequences and performed a Bayesian phylogenetic reconstruction using beast2 (Bouckaert et al., 2014). We set the crown root age at 505 Ma (Davison et al., 2015), which is consistent with fossil data and previous dated molecular phylogenies (Lutzoni et al., 2018; Strullu‐Derrien et al., 2018). We also used the youngest (437 Ma) and oldest (530 Ma) crown age estimates from Lutzoni et al. (2018) in diversification analyses that may be particularly sensitive to absolute dates.

### 2.2 Delineation into evolutionary units

We considered several ways to delineate Glomeromycotina species based on the SSU rRNA gene. In addition to the VT species proxy, we delineated Glomeromycotina *de novo* into evolutionary units (EUs) using a monophyly criterion and five different thresholds of sequence similarity ranging from 97% to 99%. We gathered Glomeromycotina sequences of the SSU rRNA gene from MaarjAM, mainly amplified by the primer pair NS31–AML2 (variable region; Lee et al., 2008; Simon et al., 1992; data set 1, Table S1). There were 36,411 sequences corresponding to 27,728 haplotypes. We first built a phylogenetic tree of these haplotypes and then applied to this tree our own algorithm of EU delineation (R‐package RPANDA; Morlon et al., 2016; R Core Team, 2020) that traverses the tree from the root to the tips, at every node computes the average similarity of all sequences descending from the node, and collapses the sequences into a single EU if their sequence dissimilarity is lower than a given threshold (Methods S2). In other words, Glomeromycotina sequences are merged into the same EU if they form a monophyletic clade and if they are on average more similar than the sequence similarity threshold. Finally, we performed Bayesian phylogenetic reconstructions of the EUs using beast2, using the same crown ages as above (Methods S1).

### 2.3 Coalescent‐based species delineation analyses

Finally, we considered the Generalized Mixed Yule Coalescent method (GMYC; Fujisawa & Barraclough, 2013; Pons et al., 2006), a species delineation approach that does not require specifying an arbitrary similarity threshold. GMYC estimates the time *t* in a reconstructed calibrated tree that separates species diversification (Yule process—before *t*) and intraspecific differentiation (coalescent process—after *t*). GMYC is too computationally intensive to be applied to the 36,411 SSU sequences; we used it here on three clades of manageable size (the family Claroideoglomeraceae; the order Diversisporales; and an early‐diverging clade composed of the orders Archaeosporales and Paraglomerales) to (i) investigate whether the SSU gene evolves fast enough to accumulate substitutions between Glomeromycotina speciation events (Bruns et al., 2018) and (ii) evaluate the biological relevance of the VT and various EU delineations. For each clade, we reconstructed Bayesian phylogenetic trees of haplotypes (Methods S1). We then ran GMYC analyses (splits R‐package; Ezard et al., 2009)) on each of these trees and evaluated the support of the GMYC model compared to a null model in which all tips are assumed to be different species, using a likelihood ratio test (LRT). If the LRT supports the GMYC model, different SSU haplotypes belong to the same Glomeromycotina species; that is, the SSU rRNA gene has time to accumulate substitutions between Glomeromycotina speciation events.

### 2.4 Total diversity estimates

We evaluated how thoroughly sampled our species‐level Glomeromycotina phylogenetic trees are by estimating the total number of VT and EUs using rarefaction curves and the Bayesian Diversity Estimation Software (bdes; Quince et al., 2008; Methods S3). bdes estimates the total number of species by extrapolating a sampled taxon abundance distribution at global scale (Quince et al., 2008).

### 2.5 Additional molecular markers

We explored the possibility of carrying some of our analyses using two other molecular markers: the large subunit (LSU) rRNA gene and the ITS2 region. We downloaded the Glomeromycotina LSU database of Delavaux et al. (2020) as well as the LSU sequences available in MaarjAM. We obtained a total 2044 sequences that we aligned using mafft and trimal. We retained the 1760 unique haplotypes, reconstructed the phylogenetic tree of the LSU sequences using beast2 and used the resulting calibrated tree to delineate Glomeromycotina LSU units with the GMYC model (same pipeline as above). We similarly downloaded the Glomeromycotina ITS data set of Lekberg et al. (2018). We tried to align them but confirmed that the ITS sequences of Glomeromycotina are very difficult to align, making them unsuitable for phylogenetic reconstruction and subsequent diversification analyses (Figure S1).

### 2.6 Diversification analyses

Unless specified otherwise, our diversification analyses were performed using the SSU rRNA gene. To account for various sources of uncertainties in the SSU rRNA data, we replicated all our diversification analyses across different species delineations, phylogenetic reconstructions and dating, and total diversity estimates. For each species delineation criterion, we obtained a consensus tree and selected 12 trees equally spaced in four independent Bayesian chains, hereafter referred to as the replicate trees. When the 12 trees were not sufficient to make a conclusion, we used 100 replicate trees.

We estimated lineage‐specific speciation rates using ClaDS, a Bayesian diversification model that accounts for speciation rate heterogeneity by modelling small rate shifts at speciation events (Maliet et al., 2019). At each speciation event, the descending lineages inherit new speciation rates sampled from a log‐normal distribution with an expected value log[*α *× *λ*] (where *λ* represents the parental speciation rate and *α* is a trend parameter) and a standard deviation *σ*. We considered the model with constant turnover *ε* (i.e., constant ratio between extinction and speciation rates; *ClaDS2*) and ran a newly developed ClaDS algorithm based on data augmentation techniques which enables us to estimate mean rates through time (Maliet & Morlon, 2022). We ran ClaDS with three independent chains, checked their convergence using a Gelman–Rubin diagnostic criterion (Gelman & Rubin, 1992) and recorded lineage‐specific speciation rates. We also recorded the estimated hyperparameters (*α*, *σ*, *ε*) and the value *m* = α × exp(*σ* ^2^/2), which indicates the general trend of the rate through time (Maliet et al., 2019). We replicated these analyses using the LSU gene.

In addition, we applied CoMET (TESS R‐package Höhna et al., 2016; May et al., 2016), another diversification approach that does not consider rate variation across lineages, but models temporal shifts in speciation and extinction rates affecting all lineages simultaneously. CoMET is a piecewise‐constant model in a Bayesian framework. We chose the Bayesian priors according to maximum likelihood estimates from treepar (Stadler, 2011), disallowed or not mass extinction events, and ran the Markov chain Monte Carlo (MCMC) chains until convergence (minimum effective sample sizes of 500).

We also fitted a series of time‐dependent and environment‐dependent birth–death diversification models using rpanda (Condamine et al., 2013; Morlon et al., 2016) to confirm the observed temporal trends and test the influence of temperature, *p*CO_2_ and land plant fossil diversity on rates of Glomeromycotina speciation. For the time‐dependent models, we considered models with constant or exponential variation of speciation rates through time and null or constant extinction rates (*fit_bd* function). As extinction is notoriously hard to estimate from reconstructed phylogenies (Rabosky, 2016), we tested the robustness of the inferred temporal trend in speciation when fixing arbitrarily high levels of extinction (Methods S4). For the environment‐dependent models, we considered an exponential dependency of the speciation rates with the environmental variable (env), namely speciation rate = b*exp(a*env), where *a* and *b* are two parameters estimated by maximum likelihood (*fit_env* function). Best‐fit models were selected based on the corrected Akaike information criterion (AICc), considering that a difference of 2 in AICc indicates that the model with the lowest AICc is better. We replicated these analyses using the LSU gene.

The influence of temperature was tested on the complete Glomeromycotina phylogenetic trees, using estimates of past global temperature (Royer et al., 2004). We also carried out a series of simulation analyses to test the robustness of our temperature‐dependent results (Methods S5). The influence of *p*CO_2_ (Foster et al., 2017) and of land plant fossil diversity was tested starting from 400 Ma, as these environmental data are not available for more ancient times. For these analyses we sliced the phylogenies at 400 and 200 Ma, and applied the diversification models to the sliced subtrees larger than 50 tips. Estimates of land plant diversity were obtained using all available Embryophyta fossils from the Paleobiology database (https://paleobiodb.org) and using the shareholder quorum subsampling method (Methods S6; Alroy, 2010).

We considered missing species in all our diversification analyses by imputing sampling fractions, computed as the number of observed VT or EUs divided by the corresponding bdes estimates of global Glomeromycotina diversity (Table 1). We used a global sampling fraction for all Glomeromycotina, as the main Glomeromycotina clades had a similar sampling fraction (Table S2). To assess the robustness of our results to global diversity estimates, we replicated all diversification analyses using a range of lower sampling fractions (from 90% to 50%; i.e., assuming that only that percentage of the global Glomeromycotina species diversity is in fact represented in our data set).

**TABLE 1:** Estimation of the total diversity of Glomeromycotina

Species delineation	Observed number of units	Estimated number of units	Sampling fraction (%)	95% confidence interval	
				Lower boundary	Upper boundary
VT	384	403	95	391 (98%)	422 (91%)
EU97	182	187	97	183 (99%)	194 (94%)
EU97.5	340	357	95	348 (98%)	370 (92%)
EU98	641	677	95	663 (97%)	695 (92%)
EU98.5	1190	1268	94	1247 (95%)	1293 (92%)
EU99	2647	2852	93	2817 (94%)	2890 (92%)

Note

Estimated sampling fraction using the Bayesian Diversity Estimation Software (bdes; Quince et al., 2008) for the different species delineations (VT or EU) assuming a Sichel species abundance distribution. The estimated number of units corresponds to the median value and we indicate the 95% confidence interval. We indicate the sampling fractions for each delineation, computed as the number of observed VT or EUs divided by the corresponding bdes estimates of global Glomeromycotina diversity. The Sichel distribution was selected compared to other distributions (log‐normal, log‐Student and inverse gaussian) based on the lowest deviance information criterion (DIC).

### 2.7 Testing for correlates of present‐day Glomeromycotina speciation rates

To further investigate the potential factors correlating with Glomeromycotina speciation rates, we assessed the relationship between lineage‐specific estimates of present‐day speciation rates (obtained with the ClaDS analyses) and characteristics of each Glomeromycotina taxonomic unit (i.e., VT or EUs).

First, to assess the effect of specialization on speciation rates, we characterized Glomeromycotina relative niche width using a set of 10 abiotic and biotic variables recorded in the MaarjAM database for each Glomeromycotina unit. In brief, among a curated data set containing Glomeromycotina sequences occurring only in natural ecosystems (data set 2; Table S2; Perez‐Lamarque et al., 2020), for each Glomeromycotina unit, we reported the number of continents, ecosystems, climatic zones, biogeographical realms, habitats and biomes where it was sampled, as well as its number of plant partners, their phylogenetic diversity and its centrality in the plant–fungus bipartite network, and performed a principal component analysis (PCA; Methods S7). For Glomeromycotina units represented by at least 10 sequences, we tested whether these PCA components reflecting Glomeromycotina niche widths were correlated with the present‐day speciation rates using both linear mixed‐models (not accounting for phylogeny) or MCMCglmm models (Hadfield, 2010). For MCMCglmm, we assumed a Gaussian residual distribution, included the fungal phylogenetic tree as a random effect, and ran the MCMC chains for 1,300,000 iterations with a burn‐in of 300,000 and a thinning interval of 500.

Next, we tested the relationship between speciation rates and geographical characteristics of Glomeromycotina units. To evaluate the effect of latitude on speciation rates, we associated each Glomeromycotina unit with its set of latitudes and used a similar MCMCglmm with an additional random effect corresponding to the Glomeromycotina unit. To account for inhomogeneous sampling along the latitudinal gradient, we reran the model on jackknifed data sets (we resampled 1,000 interactions per slice of latitude of 20°). Similarly, we tested the effect of climatic zone and habitat on speciation rates.

Then, to assess the effect of dispersal capacity on speciation rates, we evaluated the relationship between spore size and speciation rate for the few (*n* = 32) VT that contain sequences of morphologically characterized Glomeromycotina isolates (Davison et al., 2018). We gathered measures of their average spore length (Davison et al., 2018) and tested their relationship with speciation rate by using a phylogenetic generalized least square (PGLS) regression.

Finally, as a first attempt at connecting Glomeromycotina macroevolutionary diversification to microevolutionary processes, we measured intraspecific genetic diversities across Glomeromycotina units. For each Glomeromycotina unit containing at least 10 sequences, we computed genetic diversity using Tajima's estimator (θπ; Tajima, 1983; Methods S8). Using similar statistical tests as above, we investigated the correlation of Glomeromycotina genetic diversity with speciation rate, niche width, geographical characteristics and spore size. We tested the robustness of the results to the minimal number of sequences per Glomeromycotina unit (10, 15 or 20) used to compute genetic diversity and to perform the PCA.

These statistical models were replicated on the different phylogenetic trees (consensus or replicates) for each delineation and we report *p*‐values corresponding to two‐sided tests.

### 2.8 Simulation analyses

The use of a short and slowly evolving gene such as the central region of the SSU rRNA gene to delineate species may lead to an artificial lumping of species into the same unit that would reduce the number of phylogenetic branching events toward the present and result in a biased inference of temporal diversification dynamics, including an artefactual detection of a diversification slowdown (Moen & Morlon, 2014). We used simulations mimicking the evolution of the SSU rRNA gene as Glomeromycotina diversified to quantify this potential bias.

We simulated the diversification of a clade of species in the last 505 Ma, according to two scenarios: (i) constant speciation rate and no extinction and (ii) constant speciation and extinction rates (Figure S2a). To model intraspecific differentiation, we added intraspecific splits on these simulated species trees by grafting coalescent events at each tip: for each species, we uniformly sampled between two and 15 individuals and we considered that all these individuals had to coalesce before the last speciation event; the age of the coalescent tree within each species was uniformly sampled between 0 and the age of the last speciation event (with a maximum of 30 Ma). We used the functions *pbtree* and *rcoal* from the R‐packages phytools and ape (Paradis et al., 2004; Revell, 2012) to simulate the species phylogenies and the intraspecific coalescences respectively. We used two net diversification rates (*r* = 0.010 and *r* = 0.015) for simulating the species phylogenies, in order to reach a total number of species similar to that obtained with our empirical data when using the VT and EU99 (using a threshold of 99%) delineations, respectively. Next, we simulated the evolution of short 520‐bp DNA sequences on the obtained trees, using the function *simulate_alignment* (R‐package HOME; Perez‐Lamarque & Morlon, 2019). We used a substitution rate of 0.001 events per Myr and only 25% of variable sites, which resulted in an alignment that mimicked the Glomeromycotina SSU rDNA alignment. We performed 10 simulations per scenario. For each of these simulations we kept the unique haplotypes at present and applied the same pipelines as above, using the EU99 species delineation criteria: after delineating the EU99 units, we reconstructed the EU99 phylogenetic trees, ran the ClaDS analyses on these trees and recorded mean estimated speciation rates at present and 50, 100 and 150 Ma.

## 3 RESULTS

### 3.1 Glomeromycotina species delineations and phylogenetic reconstructions

We automatically delineated Glomeromycotina into EUs using a monophyly criterion and several thresholds of SSU rRNA sequence similarity (from 97% to 99%). The EU97.5 and EU98 delineations (obtained using a threshold of 97.5% and 98% respectively) provided a number of Glomeromycotina units (340 and 641) relatively comparable to the 384 currently recognized VT, while the EU97 delineation had many fewer units (182). Conversely, the EU98.5 and EU99 delineations yielded a much larger number of Glomeromycotina units (1190 and 2647). These numbers obtained with the EU98.5 and EU99 delineations were consistent with the numbers obtained using GMYC analyses, which delineate species‐like units based on detecting when splitting events in the haplotype tree start to follow branching patterns consistent with intraspecific differentiations (i.e., coalescent patterns) instead of speciation events (i.e., birth–death patterns; Tables S3‐S5). The GMYC results therefore support the idea that some VT might lump together several cryptic species (Bruns et al., 2018; Note S1), and that a 98.5% or 99% similarity threshold is more relevant for Glomeromycotina species delineation. In addition, the GMYC model is significantly supported over the model in which all SSU rRNA haplotypes correspond to a different species (GMYC LRT: *p* < .05; Figure S3), with on average 10 SSU haplotypes per species‐like unit, and a mean intraspecific sequence similarity of 99% (Table S5 and Figure S3). This indicates that the region of the SSU marker used to characterize Glomeromycotina evolves fast enough to accumulate substitutions between Glomeromycotina speciation events, meaning that it is an informative (although not perfect) marker for delineating Glomeromycotina species‐like units. In comparison, the same pipeline carried on the LSU database delineated only 181 GMYC units, suggesting that it was much less complete than the SSU database. We replicated the subsequent diversification analyses using the LSU region, even though we put more trust in our results using the SSU database given the incompleteness of the LSU database.

**FIGURE 1:**
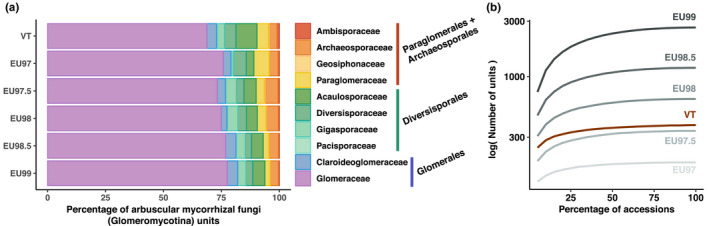
Molecular‐based species delineations of Glomeromycotina (arbuscular mycorrhizal fungi) give consistent results and indicate a nearly complete sampling. We compared the virtual taxa (VT) delineation from Öpik et al. (2010) with newly developed automatic delineations into evolutionary units (EUs) based on an average threshold of similarity and a criterion of monophyly. (a) The proportion of Glomeromycotina units (VT or EUs) in each Glomeromycotina family reveals constant proportions across delineations, although Glomeraceae tend to be relatively less abundant compared with the other Glomeromycotina family in the VT delineation. The main Glomeromycotina orders are indicated to the right of the charts: Paraglomerales + Archaeosporales, Diversisporales, and Glomerales (Glomeraceae + Claroideoglomeraceae). (b) Rarefaction curves indicating the number of Glomeromycotina units as a function of the percentage of sampled Glomeromycotina accessions revealed that the Glomeromycotina sampling in MaarjAM is close to saturation for all delineations (VT or EUs). Rarefactions were performed 100 times every 5% and the median of 100 replicates is represented

Rarefaction curves as well as bdes and Chao2 estimates of diversity suggested that more than 90% of the total Glomeromycotina diversity is represented in our SSU data set regardless of the delineation threshold (Figure 1, Table 1; Tables S5 and S6), which is consistent with the proportion of new Glomeromycotina units detected in recent studies (Sepp et al., 2019).

**FIGURE 2:**
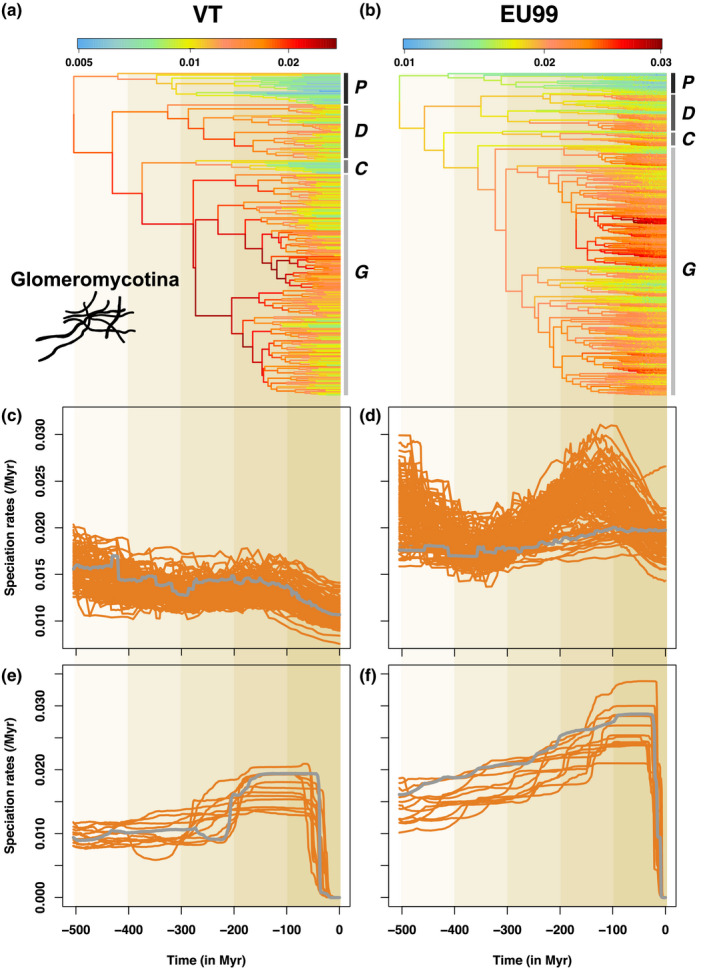
The speciation dynamics of Glomeromycotina (arbuscular mycorrhizal fungi) varies significantly through time and between lineages. (a,b) Glomeromycotina consensus phylogenetic trees corresponding to the VT (a) and EU99 (b) species delineations. Branches are coloured according to the lineage‐specific speciation rates estimated by ClaDS using the bdes estimated sampling fraction: lineages with low and high speciation rates are represented in blue and red, respectively. The main Glomeromycotina clades are indicated with the following letters: *P* = Paraglomerales + Archaeosporales, *D* = Diversisporales, *C* = Claroideoglomeraceae and *G* = Glomeraceae. (c,d) Mean speciation rates through time estimated by ClaDS, for the VT (c) and EU99 (d) delineations and using the bdes estimated sampling fraction. The mean speciation rate corresponds to the maximum a posteriori (MAP) of the mean speciation rate across all fungal lineages back in time (including extinct and unsampled lineages). Orange and grey lines represent the independent replicate trees and the consensus tree, respectively: because some of the 12 replicate trees showed different trends, we replicated ClaDS inferences using 100 replicate trees. Unlike most replicate trees, the EU99 consensus tree tends to present a limited decline in speciation rates, which reinforces the idea that consensus trees can be misleading (Janzen & Etienne, 2017). (e,f) Mean speciation rates through time estimated by CoMET, for the VT (e) and EU99 (f) delineations and using the bdes estimated sampling fraction. Orange and grey lines represent the 12 independent replicate trees and the consensus tree, respectively

The reconstructed Bayesian phylogenetic trees based on VT and EU delineations did not yield high support for the nodes separating the main Glomeromycotina orders; yet, the trees had no significantly supported conflicts either, and similar branching times of the internal nodes overall (Figure 2; Figure S4). As expected, finer delineations resulted in an increase in the number of nodes close to the present (Figure S5). However, we observed a slowdown in the accumulation of new lineages close to the present in all lineage through time plots (LTTs), including those with the finest delineations (EU98.5 and EU99; Figure S6).

### 3.2 Temporal diversification dynamics

We found that speciation rates for Glomeromycotina ranged from 0.005 to 0.03 events per lineage per Myr, using both the VT and EU SSU rRNA delineations (Figure 2; Figure S7). Speciation rates varied both within and among Glomeromycotina orders, with Glomerales and Diversisporales having the highest present‐day speciation rates (Figure S8). As expected we observed higher present‐day speciation rates for finer delineations, but at the haplotype level (i.e., at the level of the individual SSU rRNA sequences within each unit) we found a significant correlation of present‐day speciation rates computed with ClaDS using different delineations (Figure S9). Whatever the delineations, Glomeromycotina experienced their highest speciation rates between 200 and 100 Ma according to estimates obtained with ClaDS (Figure 2; Figure S10) and between 150 and 50 Ma according to CoMET (Figure 2; Figure S11).

ClaDS estimates of speciation rates at 150 Ma were 26% (*SD* ± 17) higher than those at 300 Ma with the EU99 delineation. With the VT delineation, the increase was 3% (±8). The peak was even stronger using CoMET: 30 ± 20% higher at 150 Ma in comparison to 300 Ma with the EU99 delineation (71 ± 40 with the VT delineation; Figure 2).

The peak of speciation rates was followed by a decline in the recent past (Figure 2; Figure S10), as suggested by the plateauing of the LTTs. A global decline of the speciation rates through time was independently supported by ClaDS and CoMET analyses, as well as time‐dependent models in rpanda (Morlon et al., 2011; Figures S11‐S13). This speciation rate decline was robust to all species delineations, the branching process prior (Table S7), phylogenetic uncertainty and assumed sampling fractions as low as 50%, except in ClaDS analyses where the trend disappeared in some EU99 trees and for sampling fractions lower than 70% (Figures S14 and S15). We also found a period of high speciation rates between 200 and 100 Ma followed by a decline in our analyses with the LSU region, for assumed sampling fractions as low as 60% (Figure S16).

We did not find a strong signal of extinction in our analyses: the turnover rate estimated from ClaDS was generally close to zero (Figure S12b), and models including extinctions were never selected in rpanda (Figure S13). Similarly, the extinction rates estimated in piecewise‐constant models (CoMET) were not significantly different from 0 and we did not find significant support for mass extinction events (Figure S17). Yet, forcing the extinction rate to high positive values did not modify the general trend of speciation rate slowdown (Figures S18 and S19).

### 3.3 Correlates of Glomeromycotina diversification

**FIGURE 3:**
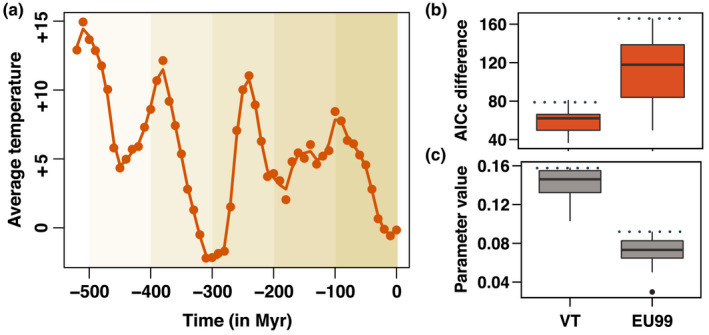
Temperature‐dependent diversification models reveal that global temperature positively associates with the speciation rates of Glomeromycotina (arbuscular mycorrhizal fungi) in the last 500 million years. (a) Average global temperature in the last 500 million years (Myr) relative to the average temperature for the period 1960–1990. The smoothed orange line represents cubic splines with 33 degrees of freedom used to fit temperature‐dependent models of Glomeromycotina diversification with rpanda. This default smoothing was estimated using the R function *smooth*.*spline*. (b) AICc difference between the best‐supported time‐dependent model and the temperature‐dependent model in rpanda, for the VT (left) and EU99 (right) delineations, using the bdes estimated sampling fraction. An AICc difference >2 indicates that there is significant support for the temperature‐dependent model. (c) Parameter estimations of the temperature‐dependent models (speciation rate ~exp[parameter * temperature]). A positive parameter value indicates a positive effect of temperature on speciation rates. For both delineations, the boxplots represent the results obtained for the consensus tree and the 12 independent replicate trees. Boxplots indicate the median surrounded by the first and third quartiles, and whiskers extend to the extreme values but no further than 1.5× the interquartile range. The horizontal dotted lines highlight the values estimated for the consensus trees. Compared to the replicate trees, the consensus trees tend to present extreme values (stronger support for the temperature‐dependent model), which reinforces the idea that consensus trees can be a misleading representation (Janzen & Etienne, 2017)

When fitting environment‐dependent models of diversification, we found that temperature‐dependent models better fit Glomeromycotina diversification than time‐dependent models, with higher speciation rates during warm climatic periods (Figure 3; Figure S20). This was true for all Glomeromycotina delineations, sampling fractions and crown ages (Figures S21‐S24), with the exception of some EU99 trees with a 50% sampling fraction (Figure S24). It was also true in our analyses using the LSU region, for sampling fractions down to 50% (Figure S29). This signal of temperature dependency was not due to a temporal trend (Figures S25 and S26) nor to an artefact caused by rate heterogeneities (Figure S27). Evidence for temperature dependency, however, decreased in some clades closer to the present, as small trees tend to be best fit by constant or time‐dependent models (Figure S28). We detected a significant positive dependency of the speciation rates on CO_2_ concentrations in some subtrees, but rarely found a significant effect of plant fossil diversity (Figure S28).

**FIGURE 4:**
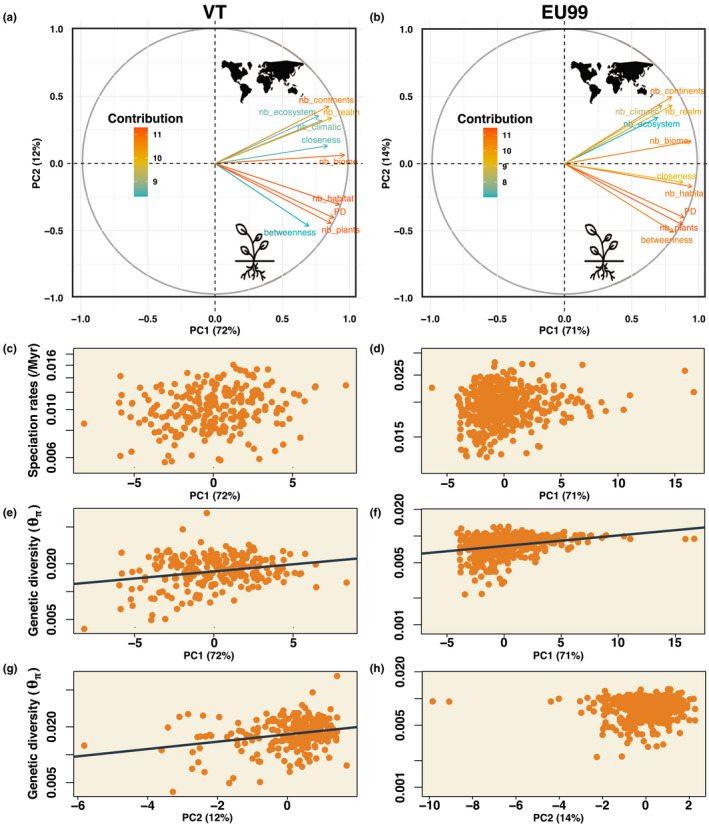
Abiotic and biotic correlates of speciation rates and genetic differentiation in Glomeromycotina (arbuscular mycorrhizal fungi). (a,b) Projection of 10 abiotic and biotic variables on the two principal components according to the VT (a) or EU99 (b) delineations. Principal component analysis (PCA) was performed for the Glomeromycotina units represented by at least 10 sequences. Colours represent the contribution of the variable to the principal components. The percentage for each principal component (PC) indicates its amount of explained variance. Tested variables were: the numbers of continents on which the Glomeromycotina unit occurs (nb_continent), of realms (nb_realm), of ecosystems (nb_ecosystems), of habitats (nb_habitats), of biomes (nb_biomes) and climatic zones (nb_climatic) (Öpik et al., 2010), as well as information about the associated plant species of each unit, such as the number of plant partners (nb_plants), the phylogenetic diversity of these plants (PD), and the betweenness and closeness measurement of each fungal unit in the plant–fungus interaction network (see Methods). (c,d) Speciation rates as a function of the PC1 component for each VT (c) or EU99 (d) unit. Only the Glomeromycotina consensus tree is represented here (other replicate trees are presented in Figure S33). (e–h) Genetic diversity (Tajima's θπ estimator) as a function of the PC1 (e,f) or PC2 (g,h) components for each VT (e–g) or EU99 (f–h) unit. Only the Glomeromycotina consensus tree is represented here (other replicate trees are presented in Figure S33). The grey lines indicate the statistically significant linear regression between the two variables inferred using MCMCglmm

The PCA of Glomeromycotina relative niche width characteristics had a first principal component (PC1) that indicated the propensity of each Glomeromycotina unit (VT or EUs) to be vastly distributed among continents, ecosystems and/or associated with many plant species and lineages (i.e., high generalism), whereas the second principal component (PC2) indicated the propensity of a given Glomeromycotina unit to associate with few plant species on many continents (i.e., high specialism toward plants; Figures S30‐S32). Hence, PC1 reflects Glomeromycotina niche width, whereas PC2 discriminates the width of the abiotic relative to the biotic niche (Figure 4a,b). We found a positive correlation between lineage‐specific speciation rates and PC1 in the majority of the VT and EU99 trees, but no significant correlation with PC2 (Figure 4c,d; Figure S33a). However, these results were no longer significant when controlling for phylogenetic nonindependence between Glomeromycotina units (Figure S33b), probably because a single Glomeraceae clade, including the abundant and widespread morphospecies *Rhizophagus irregularis* and *R*. *clarus* (high PC1 values), had both the highest speciation rates and the largest niche widths among Glomeromycotina (Figure S34).

Although Glomeromycotina diversity is currently higher in the (sub)tropics (Figure S35), we found no effect of latitude on speciation rates, regardless of the Glomeromycotina delineation or the minimum number of sequences per Glomeromycotina unit (MCMCglmm: *p* > .05). In addition, we did not detect a higher total number of Glomeromycotina species in grasslands compared to forests (Figure S36; confirming the results of Davison et al., 2015), and it is thus not surprising that we reported no effect of habitat or climatic zone on speciation rates (Figure S37), suggesting that tropical grasslands are not particular diversification hotspots for Glomeromycotina. Similarly, we recovered no significant correlation between spore size and speciation rate (Figure S38), nor between spore size and level of endemism (Figure S39).

Finally, Tajima's estimator of Glomeromycotina genetic diversity was significantly and positively correlated with niche width (PC1) for all Glomeromycotina delineations and minimal number of sequences per Glomeromycotina unit considered, and in particular with abiotic aspects of the niche (PC2) in many cases (Figure 4e–h; Figure S33). Genetic diversity was not correlated with speciation rate (Figure S33), latitude, habitat, climatic zone (MCMCglmm: *p* > .05) or spore size (PGLS: *p* > .05).

### 3.4 Simulation results

**FIGURE 5:**
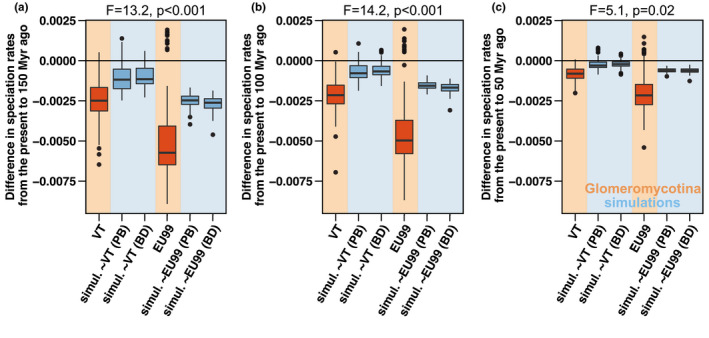
Artefactual species lumping and lack of phylogenetic resolution in the SSU rRNA region are not enough to explain the temporal decline in speciation rates detected in Glomeromycotina. Comparison of the magnitude of the decline in speciation rates observed in Glomeromycotina (in orange) and on simulated data (in blue). The intensity of the slowdown is measured as the difference between the mean speciation rate estimated at present and the mean speciation rate estimated 150 Ma (a), 100 Ma (b) or 50 Ma (c). Negative differences indicate a speciation rate decline. Sequence alignments were simulated on phylogenetic trees obtained under a scenario of constant speciation rate and no extinction (i.e., pure birth “PB”) and constant speciation and extinction rates (i.e., birth death “BD”), with characteristics mimicking the slow evolution of the SSU rRNA marker. We simulated phylogenies with two different net diversification rates, such that we obtained simulations with total numbers of species similar to the total numbers of VT or EU99 units (Figure S2). Boxplots indicate the median surrounded by the first and third quartiles, and whiskers extend to the extreme values but no further than 1.5× the interquartile range. Each boxplot represents results for the consensus trees and the 12 independent replicate trees for each of the 10 simulations, and for the consensus trees and the 100 independent replicate trees for the Glomeromycotina. Differences between the magnitude of the decline measured in Glomeromycotina (VT or EU99) and in the corresponding simulations were tested using linear models (reported at the top of the plots)

When we simulated the evolution of a short DNA gene mimicking the SSU rRNA marker and used it to delineate species, we found that the number of EU99‐delineated units was generally lower than the number of simulated species (~10% to 20% lower; Figure S2b). Hence, even the EU99 delineation tends to lump together some closely related species. As expected, this lumping resulted in an artefactual inference of a decline of speciation rates toward the present, but this artefactual decline was significantly smaller in magnitude than that observed in Glomeromycotina (Figure 5). Hence, these analyses suggest that the lumping of species resulting from the use of a small, slowly evolving marker is unlikely to fully explain the strong temporal decline in speciation rate we found in Glomeromycotina.

## 4 DISCUSSION

### 4.1 Glomeromycotina species delineations, diversity and phylogeny

It is difficult to delineate species in Glomeromycotina, which are poorly differentiated morphologically and mainly characterized by environmental sequences (Bruns et al., 2018). Our GMYC analyses suggest that Glomeromycotina species‐like units correspond to SSU rRNA haplotypes with a sequence similarity of 98.5–99%. With this criterion of species delineation, we estimate that there are between 1300 and 2900 Glomeromycotina “species.” These estimates are largely above the number of currently described morphospecies or VT (Note S1) but remain low in comparison with other fungal groups, such as the Agaricomycetes that include taxa forming ectomycorrhiza (Varga et al., 2019).

Our phylogenies based on the SSU rRNA gene did not resolve the branching of the Glomeromycotina orders, with node support values similar to those of previous studies (Davison et al., 2015; Krüger et al., 2012; Rimington et al., 2018; Note S2). These findings confirm that additional genomic evidence is required to reach consensus. We considered this uncertainty in species delineation and phylogenetic reconstruction by repeating our diversification analyses across species delineation criteria and on a set of trees spanning the probable tree space. We found effects of species delineation consistent with a priori expectations: criteria that lump together more dissimilar sequences (e.g., those that use a lower percentage of similarity cut‐off) result in lower diversity estimates, lower estimates of speciation rates and patterns of diversification through time that reflect longer terminal branch‐lengths, such as peaks of diversification that occur earlier. Despite this variability, we found that general patterns, such as the observed temporal decline in speciation rates and the significant association between temperature and speciation rates, were consistent across species delineations and trees. Therefore, our study based on a short SSU (or LSU) rRNA region should encourage both efforts to obtain more genetic data, including longer reads (Krehenwinkel et al., 2019; Tedersoo et al., 2021) and additional genomic information, with the aim of reconstructing better supported, comprehensive phylogenies, and efforts to conduct diversification analyses despite uncertainty in the data for groups where better data are not yet available.

### 4.2 Glomeromycotina diversify slowly

We found speciation rates for Glomeromycotina an order of magnitude lower than rates typically found for macro‐eukaryotes (Maliet et al., 2019; Upham et al., 2019), such as plants (Zanne et al., 2014), or Agaricomycetes (Varga et al., 2019). Low speciation rates in Glomeromycotina may be linked to their multinucleate hyphal state (Yildirir et al., 2020), to their occasional long‐distance dispersal that homogenizes populations globally over evolutionary timescales (Savary et al., 2018) and/or to the fact that they are generalist obligate symbionts (Morlon et al., 2012). Regardless of the proximal cause, and in contrast to Agaricomycetes for example, which present a large diversity of species, morphologies and ecologies, Glomeromycotina have poorly diversified in the last 500 Myr despite their ubiquity; their niche space is restricted to plant roots and the surrounding soil because of their obligate dependence on plants for more than 400 Myr (Rich et al., 2017; Tisserant et al., 2013).

Our estimates of speciation rates were highly variable across lineages. We reported the highest speciation rates in Glomeraceae and Diversisporaceae. Speciation rates in Paraglomeraceae and Archaeosporaceae, which are thought to be less beneficial for the plants than the fast diversifying Glomeraceae and Diversisporaceae (Säle et al., 2021), were an order of magnitude lower. We can therefore speculate that good symbiotic abilities may favour diversification, although this remains to be tested in further investigations.

We found little evidence for species extinction in Glomeromycotina, including at mass extinction events. Because Glomeromycotina are relatively widespread and have an ancient tendency toward generalism, they might therefore be quite resilient to land plant mass extinctions, and low extinction rates have been predicted previously based on their ecology (Morton, 1990). Yet, these low extinction rate estimates could also come from the difficulty of estimating extinction from molecular phylogenies (Rabosky, 2016), one of the limitations of phylogeny‐based diversification analyses (Note S3). Fossils of Glomeromycotina that can be ascribed to species or genera are too scarce to support or conflict with this finding.

### 4.3 Glomeromycotina diversification through time

The observed peak of Glomeromycotina speciations detected between 200 and 100 Ma (or 150–50 Ma depending on the models) was mainly linked to the frequent speciations in the largest family Glomeraceae. This peak was concomitant with the radiation of flowering plants (Sauquet & Magallón, 2018), but also with a major continental reconfiguration, including the breakdown of Pangaea and the formation of climatically contrasted landmasses (Davison et al., 2015). This period was also characterized by a warm climate potentially directly or indirectly favourable to Glomeromycotina diversification, such that disentangling the impact of these various factors on Glomeromycotina diversification rates is not straightforward. Interestingly, a peak of speciations at this period was also found in the Agaricomycetes, a clade of fungi including lineages forming ectomycorrhizae (Varga et al., 2019).

This peak in the occurrence of speciation events was followed by a decline in speciation rates. The detection of temporal declines in speciation rates in phylogenetic diversification analyses can sometimes be artefactual, for example if some species are incorrectly lumped together during species delineation or if the proportion of species not represented in the phylogeny is underestimated (Moen & Morlon, 2014). We considered these potential biases, conducted sensitivity analyses, and found that the observed slowdown was robust and even amplified under scenarios of high extinction. Some Glomeromycotina species are probably lumped together into the same SSU haplotypes (Krüger et al., 2012), but both our use of an overly small assumed sampling intensity (50%) and our simulation analyses demonstrated that this lumping is not sufficient to explain the observed slowdown. In addition, we also detected a temporal decline in speciation rates when using another marker (the LSU rRNA gene).

Temporal declines in speciation rates have been observed in many clades, including microorganisms (Condamine et al., 2019; Morlon et al., 2012; Rabosky & Lovette, 2008). They have often been interpreted as a progressive reduction of the number of available niches as species diversify and accumulate (Moen & Morlon, 2014; Rabosky, 2009). In Glomeromycotina, this potential effect of niche saturation could be exacerbated by a reduction of their niches linked to both repetitive breakdowns of their symbiosis with plants and climatic changes. Indeed, since the Cretaceous, many plant lineages evolved alternative root symbioses or became nonsymbiotic (Brundrett & Tedersoo, 2018; Maherali et al., 2016; Selosse & Le Tacon, 1998; Werner et al., 2018): ~20% of extant plants no longer interact with Glomeromycotina (van der Heijden et al., 2015). Additionally, the cooling of the Earth during the Cenozoic reduced the surface of tropical regions (Meseguer & Condamine, 2020; Ziegler et al., 2003), which tend to be a reservoir of ecological niches for Glomeromycotina (Brundrett & Tedersoo, 2018; Davison et al., 2015; Read, 1991).

The difficulty of reconstructing past symbiotic associations prevents direct testing of the hypothesis that the emergence of new root symbioses in plants led to a decline in speciation rates in Glomeromycotina. However, we tested the hypothesis that global temperature changes affected speciation rates and found a strong relationship. Such associations between temperature and speciation rates have been observed previously in eukaryotes and have several potential causes (Condamine et al., 2019). In particular, the productivity hypothesis states that resources and associated ecological niches are more numerous in warm and productive environments, especially when the tropics are large, which entail higher speciation rates (Clarke & Gaston, 2006). This hypothesis is particularly relevant for Glomeromycotina, which have many host plant niches in the tropics, as shown by their latitudinal diversity gradient, and potentially relatively less in temperate and polar regions (Toussaint et al., 2020), where a higher proportion of plants are nonmycorrhizal (Bueno et al., 2017) or ectomycorrhizal (Brundrett & Tedersoo, 2018; Varga et al., 2019). Hence, the observed effect of past global temperatures could reflect the shrinkage of tropical areas and the associated decrease of the relative proportion of arbuscular mycorrhizal plants. Future developments of diversification models incorporating interspecific interactions would allow us to better test these hypotheses.

A few Glomeromycotina clades displayed significant support for diversification models with a positive dependency of speciation rates on CO_2_ concentrations, which reinforces the idea that for the corresponding Glomeromycotina, benefits retrieved from plants could have been amplified by high CO_2_ concentrations and fostered diversification (Field et al., 2016; Humphreys et al., 2010). Conversely, we found a limited effect of land plant fossil diversity, which indicates that variations in the tempo of Glomeromycotina diversification did not systematically follow those of land plants. Still, the possible concordance of the peak of Glomeromycotina speciations with the radiation of the Angiosperms is noteworthy, in particular in Glomeraceae that frequently interact with present‐day Angiosperms (Rimington et al., 2018). Plant diversification might have fostered the diversification of Glomeromycotina from the emergence of land plants until the Mesozoic (Lutzoni et al., 2018; Morton, 1990), but less so thereafter, when Glomeromycotina diversification declined while some flowering plants radiated, including Glomeromycotina‐associated groups, such as the Poaceae, but also Glomeromycotina‐free groups such as the extraordinary radiation of Orchidaceae (Givnish et al., 2015), blurring codiversification patterns (Figure S40; Cleal & Cascales‐Miñana, 2014; Ramírez‐Barahona et al., 2020).

### 4.4 Correlates of Glomeromycotina recent speciation rates

Looking at the correlates of Glomeromycotina present‐day speciation rates, we found no effect of habitat or climatic zone, even though Glomeromycotina are more frequent and diverse in the tropics (Davison et al., 2015; Pärtel et al., 2017; Toussaint et al., 2020) and we reported a positive correlation with global temperature. Further work, including a more thorough sampling of the distribution of Glomeromycotina species across latitudes and habitats, would be required to confirm these patterns and to distinguish whether speciation events are indeed no more frequent in the tropics or, if they are, whether long‐distance dispersal redistributes the new lineages at different latitudes over long timescales. Contrary to previous predictions (Pärtel et al., 2017), we did not find that tropical grasslands are diversification hotspots for Glomeromycotina; in fact we did not find higher Glomeromycotina species richness in (tropical) grasslands vs. forests at a global scale, in agreement with Davison et al. (2015).

Similarly, although the temporal changes in the availability of Glomeromycotina niches probably influenced the diversification of the group, we found little support for Glomeromycotina species with larger niche width having higher lineage‐specific speciation rates. We also note that there are important aspects of the niche that we do not (and cannot yet) account for in our characterization of Glomeromycotina niche width: it is thought that some Glomeromycotina species may mainly provide mineral nutrients extracted from the soil, whereas others may be more specialized in protecting plants from biotic or abiotic stresses (Chagnon et al., 2013) and such (inter‐ or intraspecific) functional variations may have evolutionary significance. Finally, although spore size is often inversely related to dispersal capacity (Nathan et al., 2008), which can limit speciation by increasing gene flow, we found no significant correlation between spore size and speciation rates, which may be explained either by a weak or absent effect or by the low number of species for which data are available. In addition, the absence of a correlation between spore size and level of endemism suggests that even Glomeromycotina with large spores experience long‐distance dispersal (Davison et al., 2018; Kivlin, 2020). Thus, although large spores might limit dispersal at smaller (e.g., intracontinental) scales in Glomeromycotina (Bueno & Moora, 2019; Chaudhary et al., 2020), this does not seem to affect speciation rates.

In Glomeromycotina, intraspecific variability is an important source of functional diversity (Munkvold et al., 2004; Savary et al., 2018) and their genetic diversity may indicate the intraspecific variability on which selection can act, potentially leading to speciation. Here, geographically widespread Glomeromycotina species appear to be more genetically diverse, as previously suggested by population genomics (Savary et al., 2018), but do not necessarily speciate more frequently. Along with a decoupling between genetic diversity and lineage‐specific speciation rate, this suggests that the accumulation of genetic diversity in the SSU region among distant subpopulations is not enough to spur Glomeromycotina speciation.

### 4.5 Analysing diversification dynamics using a short marker gene

Short DNA regions, like those used in metabarcoding surveys, typically do not allow us to robustly delineate species, estimate global‐scale diversity or reconstruct phylogenetic trees. As these three aspects can all affect results of diversification analyses (Moen & Morlon, 2014), such analyses are rarely performed with these types of data. Yet, for many species‐rich groups of organisms, in particular microorganisms, no other data currently provide a thorough representation of diversity at the “species” level. Hence, these data, although far from ideal, are the only ones that can be used to study the past diversification of such groups (see Lewitus et al., 2018; Louca et al., 2018 for antecedents). The approach we took here is to recognize all these potential sources of uncertainty and biases and to test the robustness of our results. We demonstrated the usefulness of this approach: while some results inevitably depend on the choices made for species delineation, phylogenetic reconstruction and the estimation of global scale diversity, others are sufficiently strong to hold despite uncertainty in the data. Our results therefore illustrate that using a short DNA marker (e.g., a metabarcode) combined with intensive sensitivity analyses can be useful for studying the diversification dynamics of poorly known groups.

## 5 CONCLUSION

Our findings that Glomeromycotina have low speciation rates, probably constrained by the availability of suitable niches, reinforce the vision of Glomeromycotina as an “evolutionary cul‐de‐sac” (Malloch, 1987). We interpret the significant decline in speciation rates toward the present as the conjunction of the emergence of plant lineages not associated with Glomeromycotina and the reduction of tropical areas induced by climate cooling, in the context of obligate dependence of Glomeromycotina on plants. Temporal declines in speciation rates have often been interpreted as the signal of adaptive radiations (Harmon et al., 2003; Moen & Morlon, 2014), that is clades that have experienced a rapid accumulation of morphological, ecological and species diversity (Simpson, 1953). Conversely, Glomeromycotina provide here a striking example of a clade with slow morphological, ecological and species diversification that features a pattern of temporal decline in speciation rates, which might reflect the reduction of the global availability of their mycorrhizal niches.

## AUTHOR CONTRIBUTIONS

All the authors designed the study. M.Ö. gathered the data and B.P.L. performed the analyses. O.M. and A.C.A.S. provided some of the code. B.P.L. and H.M. wrote the first version of the manuscript and all authors contributed substantially to the revisions.

## CONFLICTS OF INTEREST

The authors declare that there are no conflicts of interest.

## Supporting information

Supplementary Material

## Data Availability

All of the data used in this study are available in the open‐access MaarjAM database (https://maarjam.botany.ut.ee). Spore lengths of Glomeromycotina were collected in the supplementary data of Davison et al. (2018). New scripts for delineating evolutionary units (EU) are available in the R‐package RPANDA (through this GitHub branch for the moment: https://github.com/hmorlon/PANDA/tree/Benoit_phylosignal). The alignment of all the SSU rRNA sequences and their associated metadata is publicly accessible through the Open Science Framework (osf) portal: osf.io/y2ts5.

## References

[mec16478-bib-0001] Alroy, J. (2010). Geographical, environmental and intrinsic biotic controls on Phanerozoic marine diversification. Palaeontology, 53(6), 1211–1235. 10.1111/j.1475-4983.2010.01011.x

[mec16478-bib-0002] Barnosky, A. D. (2001). Distinguishing the effects of the red queen and court jester on miocene mammal evolution in the northern rocky mountains. Journal of Vertebrate Paleontology, 21(1), 172–185.

[mec16478-bib-0003] Benton, M. J. (2009). The Red Queen and the Court Jester: Species diversity and the role of biotic and abiotic factors through time. Science, 323(5915), 728–732. 10.1126/science.1157719 19197051

[mec16478-bib-0004] Bouckaert, R. , Heled, J. , Kühnert, D. , Vaughan, T. , Wu, C.‐H. , Xie, D. , Suchard, M. A. , Rambaut, A. , & Drummond, A. J. (2014). BEAST 2: A software platform for Bayesian evolutionary analysis. PLoS Computational Biology, 10(4), e1003537. 10.1371/journal.pcbi.1003537 24722319 PMC3985171

[mec16478-bib-0005] Bredenkamp, G. J. , Spada, F. , & Kazmierczak, E. (2002). On the origin of northern and southern hemisphere grasslands. Plant Ecology, 163(2), 209–229. 10.1023/A:1020957807971

[mec16478-bib-0006] Brundrett, M. C. , & Tedersoo, L. (2018). Evolutionary history of mycorrhizal symbioses and global host plant diversity. New Phytologist, 220(4), 1108–1115. 10.1111/nph.14976 29355963

[mec16478-bib-0007] Bruns, T. D. , Corradi, N. , Redecker, D. , Taylor, J. W. , & Öpik, M. (2018). Glomeromycotina: What is a species and why should we care? New Phytologist, 220(4), 963–967. 10.1111/nph.14913 29165821

[mec16478-bib-0008] Bueno, C. G. , & Moora, M. (2019). How do arbuscular mycorrhizal fungi travel? New Phytologist, 222(2), 645–647. 10.1111/nph.15722 30895649

[mec16478-bib-0009] Bueno, C. G. , Moora, M. , Gerz, M. , Davison, J. , Öpik, M. , Pärtel, M. , Helm, A. , Ronk, A. , Kühn, I. , & Zobel, M. (2017). Plant mycorrhizal status, but not type, shifts with latitude and elevation in Europe. Global Ecology and Biogeography, 26(6), 690–699. 10.1111/geb.12582

[mec16478-bib-0010] Chagnon, P.‐L. , Bradley, R. L. , Maherali, H. , & Klironomos, J. N. (2013). A trait‐based framework to understand life history of mycorrhizal fungi. Trends in Plant Science, 18(9), 484–491. 10.1016/j.tplants.2013.05.001 23756036

[mec16478-bib-0011] Chaudhary, V. B. , Nolimal, S. , Sosa‐Hernández, M. A. , Egan, C. , & Kastens, J. (2020). Trait‐based aerial dispersal of arbuscular mycorrhizal fungi. New Phytologist, 228(1), 238–252. 10.1111/nph.16667 32421866

[mec16478-bib-0012] Chomicki, G. , Kiers, E. T. , & Renner, S. S. (2020). The evolution of mutualistic dependence. Annual Review of Ecology, Evolution, and Systematics, 51(1), 409–432. 10.1146/annurev-ecolsys-110218-024629

[mec16478-bib-0013] Clarke, A. , & Gaston, K. J. (2006). Climate, energy and diversity. Proceedings of the Royal Society B: Biological Sciences, 273(1599), 2257–2266. 10.1098/rspb.2006.3545 PMC1636092 16928626

[mec16478-bib-0014] Cleal, C. J. , & Cascales‐Miñana, B. (2014). Composition and dynamics of the great Phanerozoic Evolutionary Floras. Lethaia, 47(4), 469–484. 10.1111/let.12070

[mec16478-bib-0015] Condamine, F. L. , Rolland, J. , Höhna, S. , Sperling, F. A. H. , & Sanmartín, I. (2018). Testing the role of the Red Queen and Court Jester as drivers of the macroevolution of Apollo butterflies. Systematic Biology, 67(6), 940–964. 10.1093/sysbio/syy009 29438538

[mec16478-bib-0016] Condamine, F. L. , Rolland, J. , & Morlon, H. (2013). Macroevolutionary perspectives to environmental change. Ecology Letters, 16(SUPPL.1), 72–85. 10.1111/ele.12062 23331627

[mec16478-bib-0017] Condamine, F. L. , Rolland, J. , & Morlon, H. (2019). Assessing the causes of diversification slowdowns: temperature‐dependent and diversity‐dependent models receive equivalent support. Ecology Letters, 22(11), 1900–1912. 10.1111/ele.13382 31486279

[mec16478-bib-0018] Correia, M. , Heleno, R. , da Silva, L. P. , Costa, J. M. , & Rodríguez‐Echeverría, S. (2019). First evidence for the joint dispersal of mycorrhizal fungi and plant diaspores by birds. New Phytologist, 222(2), 1054–1060. 10.1111/nph.15571 30372538

[mec16478-bib-0019] Davison, J. , Moora, M. , Öpik, M. , Adholeya, A. , Ainsaar, L. , Bâ, A. , Burla, S. , Diedhiou, A. G. , Hiiesalu, I. , Jairus, T. , Johnson, N. C. , Kane, A. , Koorem, K. , Kochar, M. , Ndiaye, C. , Pärtel, M. , Reier, Ü. , Saks, Ü. , Singh, R. , … Zobel, M. (2015). Global assessment of arbuscular mycorrhizal fungus diversity reveals very low endemism. Science, 349(6251), 970–973. 10.1126/science.aab1161 26315436

[mec16478-bib-0020] Davison, J. , Moora, M. , Öpik, M. , Ainsaar, L. , Ducousso, M. , Hiiesalu, I. , Jairus, T. , Johnson, N. , Jourand, P. , Kalamees, R. , Koorem, K. , Meyer, J.‐Y. , Püssa, K. , Reier, Ü. , Pärtel, M. , Semchenko, M. , Traveset, A. , Vasar, M. , & Zobel, M. (2018). Microbial island biogeography: isolation shapes the life history characteristics but not diversity of root‐symbiotic fungal communities. The ISME Journal, 12(9), 2211–2224. 10.1038/s41396-018-0196-8 29884829 PMC6092392

[mec16478-bib-0021] Delavaux, C. S. , Sturmer, S. L. , Wagner, M. R. , Schütte, U. , Morton, J. B. , & Bever, J. D. (2020). Utility of large subunit for environmental sequencing of arbuscular mycorrhizal fungi: A new reference database and pipeline. New Phytologist, 1–5, 10.1111/nph.17080 33190292

[mec16478-bib-0022] Egan, C. , Li, D.‐W. , & Klironomos, J. (2014). Detection of arbuscular mycorrhizal fungal spores in the air across different biomes and ecoregions. Fungal Ecology, 12, 26–31. 10.1016/j.funeco.2014.06.004

[mec16478-bib-0023] Ezard, T. , Fujisawa, T. , & Barraclough, T. G. (2009). SPLITS: SPecies’ Limits by Threshold Statistics. R‐package. https://rdrr.io/rforge/splits/. Accessed June, 2019.

[mec16478-bib-0024] Feijen, F. A. , Vos, R. A. , Nuytinck, J. , & Merckx, V. S. F. T. (2018). Evolutionary dynamics of mycorrhizal symbiosis in land plant diversification. Scientific Reports, 8(1), 10698. 10.1038/s41598-018-28920-x 30013185 PMC6048063

[mec16478-bib-0025] Field, K. J. , Pressel, S. , Duckett, J. G. , Rimington, W. R. , & Bidartondo, M. I. (2015). Symbiotic options for the conquest of land. Trends in Ecology & Evolution, 30(8), 477–486. 10.1016/j.tree.2015.05.007 26111583

[mec16478-bib-0026] Field, K. J. , Rimington, W. R. , Bidartondo, M. I. , Allinson, K. E. , Beerling, D. J. , Cameron, D. D. , Duckett, J. G. , Leake, J. R. , & Pressel, S. (2016). Functional analysis of liverworts in dual symbiosis with Glomeromycota and Mucoromycotina fungi under a simulated Palaeozoic CO_2_ decline. ISME Journal, 10(6), 1514–1526. 10.1038/ismej.2015.204 26613340 PMC5029179

[mec16478-bib-0027] Foster, G. L. , Royer, D. L. , & Lunt, D. J. (2017). Future climate forcing potentially without precedent in the last 420 million years. Nature Communications, 8(1), 14845. 10.1038/ncomms14845 PMC5382278 28375201

[mec16478-bib-0028] Fujisawa, T. , & Barraclough, T. G. (2013). Delimiting species using single‐locus data and the generalized mixed yule coalescent approach: A revised method and evaluation on simulated data sets. Systematic Biology, 62(5), 707–724. 10.1093/sysbio/syt033 23681854 PMC3739884

[mec16478-bib-0029] Gelman, A. , & Rubin, D. B. (1992). Inference from iterative simulation using multiple sequences. Statistical Science, 7(4), 457–472. 10.1214/ss/1177011136

[mec16478-bib-0030] Givnish, T. J. , Spalink, D. , Ames, M. , Lyon, S. P. , Hunter, S. J. , Zuluaga, A. , Iles, W. J. D. , Clements, M. A. , Arroyo, M. T. K. , Leebens‐Mack, J. , Endara, L. , Kriebel, R. , Neubig, K. M. , Whitten, W. M. , Williams, N. H. , & Cameron, K. M. (2015). Orchid phylogenomics and multiple drivers of their extraordinary diversification. Proceedings of the Royal Society B: Biological Sciences, 282(1814), 20151553. 10.1098/rspb.2015.1553 PMC4571710 26311671

[mec16478-bib-0031] Hadfield, J. D. (2010). MCMC methods for multi‐response generalized linear mixed models: The MCMCglmm R package. Journal of Statistical Software, 33(2), 1–22. 10.18637/jss.v033.i02 20808728

[mec16478-bib-0032] Harmon, L. J. , Schulte, J. A. , Larson, A. , & Losos, J. B. (2003). Tempo and mode of evolutionary radiation in Iguanian lizards. Science, 301(5635), 961–964. 10.1126/science.1084786 12920297

[mec16478-bib-0033] Höhna, S. , May, M. R. , & Moore, B. R. (2016). TESS: An R package for efficiently simulating phylogenetic trees and performing Bayesian inference of lineage diversification rates. Bioinformatics, 32(5), 789–791. 10.1093/bioinformatics/btv651 26543171

[mec16478-bib-0034] Humphreys, C. P. , Franks, P. J. , Rees, M. , Bidartondo, M. I. , Leake, J. R. , & Beerling, D. J. (2010). Mutualistic mycorrhiza‐like symbiosis in the most ancient group of land plants. Nature Communications, 1(8), 103. 10.1038/ncomms1105 21045821

[mec16478-bib-0035] James, T. Y. , Kauff, F. , Schoch, C. L. , Matheny, P. B. , Hofstetter, V. , Cox, C. J. , Celio, G. , Gueidan, C. , Fraker, E. , Miadlikowska, J. , Lumbsch, H. T. , Rauhut, A. , Reeb, V. , Arnold, A. E. , Amtoft, A. , Stajich, J. E. , Hosaka, K. , Sung, G.‐H. , Johnson, D. , … Vilgalys, R. (2006). Reconstructing the early evolution of Fungi using a six‐gene phylogeny. Nature, 443(7113), 818–822. 10.1038/nature05110 17051209

[mec16478-bib-0036] Janzen, T. , & Etienne, R. S. (2017). Inferring the role of habitat dynamics in driving diversification: evidence for a species pump in Lake Tanganyika cichlids. BioRxiv, 11(2), 1–18. 10.1101/085431

[mec16478-bib-0037] Katoh, K. , & Standley, D. M. (2013). MAFFT Multiple sequence alignment software version 7: Improvements in performance and usability. Molecular Biology and Evolution, 30(4), 772–780. 10.1093/molbev/mst010 23329690 PMC3603318

[mec16478-bib-0038] Kivlin, S. N. (2020). Global mycorrhizal fungal range sizes vary within and among mycorrhizal guilds but are not correlated with dispersal traits. Journal of Biogeography, 47(9), 1994–2001. 10.1111/jbi.13866

[mec16478-bib-0039] Krehenwinkel, H. , Pomerantz, A. , Henderson, J. B. , Kennedy, S. R. , Lim, J. Y. , Swamy, V. , Shoobridge, J. D. , Graham, N. , Patel, N. H. , Gillespie, R. G. , & Prost, S. (2019). Nanopore sequencing of long ribosomal DNA amplicons enables portable and simple biodiversity assessments with high phylogenetic resolution across broad taxonomic scale. GigaScience, 8(5), giz006 10.1093/gigascience/giz006 30824940 PMC6503943

[mec16478-bib-0040] Krüger, M. , Krüger, C. , Walker, C. , Stockinger, H. , & Schüßler, A. (2012). Phylogenetic reference data for systematics and phylotaxonomy of arbuscular mycorrhizal fungi from phylum to species level. New Phytologist, 193(4), 970–984. 10.1111/j.1469-8137.2011.03962.x 22150759

[mec16478-bib-0041] Lee, J. , Lee, S. , & Young, J. P. W. (2008). Improved PCR primers for the detection and identification of arbuscular mycorrhizal fungi. FEMS Microbiology Ecology, 65(2), 339–349. 10.1111/j.1574-6941.2008.00531.x 18631176

[mec16478-bib-0042] Lekberg, Y. , Vasar, M. , Bullington, L. S. , Sepp, S.‐K. , Antunes, P. M. , Bunn, R. , Larkin, B. G. , & Öpik, M. (2018). More bang for the buck? Can arbuscular mycorrhizal fungal communities be characterized adequately alongside other fungi using general fungal primers? New Phytologist, 220(4), 971–976. 10.1111/nph.15035 29388685

[mec16478-bib-0043] Lewitus, E. , Bittner, L. , Malviya, S. , Bowler, C. , & Morlon, H. (2018). Clade‐specific diversification dynamics of marine diatoms since the Jurassic. Nature Ecology and Evolution, 2(11), 1715–1723. 10.1038/s41559-018-0691-3 30349092 PMC6217985

[mec16478-bib-0044] Louca, S. , Shih, P. M. , Pennell, M. W. , Fischer, W. W. , Parfrey, L. W. , & Doebeli, M. (2018). Bacterial diversification through geological time. Nature Ecology and Evolution, 2(9), 1458–1467. 10.1038/s41559-018-0625-0 30061564

[mec16478-bib-0045] Lutzoni, F. , Nowak, M. D. , Alfaro, M. E. , Reeb, V. , Miadlikowska, J. , Krug, M. , Arnold, A. E. , Lewis, L. A. , Swofford, D. L. , Hibbett, D. , Hilu, K. , James, T. Y. , Quandt, D. , & Magallón, S. (2018). Contemporaneous radiations of fungi and plants linked to symbiosis. Nature Communications, 9(1), 1–11. 10.1038/s41467-018-07849-9 PMC6303338 30575731

[mec16478-bib-0046] Magallón, S. , & Sanderson, M. J. (2001). Absolute diversification rates in angiosperm clades. Evolution, 55(9), 1762–1780. 10.1111/j.0014-3820.2001.tb00826.x 11681732

[mec16478-bib-0047] Maherali, H. , Oberle, B. , Stevens, P. F. , Cornwell, W. K. , & McGlinn, D. J. (2016). Mutualism persistence and abandonment during the evolution of the mycorrhizal symbiosis. American Naturalist, 188(5), E113–E125. 10.1086/688675 27788343

[mec16478-bib-0048] Maliet, O. , Hartig, F. , & Morlon, H. (2019). A model with many small shifts for estimating species‐specific diversification rates. Nature Ecology & Evolution, 3(7), 1086–1092. 10.1038/s41559-019-0908-0 31160736

[mec16478-bib-0049] Maliet, O. , & Morlon, H. (2022). Fast and accurate estimation of species‐specific diversification rates using data augmentation. Systematic Biology, 71(2), 353–366. 10.1093/sysbio/syab055 34228799

[mec16478-bib-0050] Malloch, D. M. (1987). The evolution of mycorrhizae. Canadian Journal of Plant Pathology, 9, 398–402.

[mec16478-bib-0051] May, M. R. , Höhna, S. , & Moore, B. R. (2016). A Bayesian approach for detecting the impact of mass‐extinction events on molecular phylogenies when rates of lineage diversification may vary. Methods in Ecology and Evolution, 7(8), 947–959. 10.1111/2041-210X.12563

[mec16478-bib-0052] Meseguer, A. S. , & Condamine, F. L. (2020). Ancient tropical extinctions at high latitudes contributed to the latitudinal diversity gradient. Evolution, 74(9), 1966–1987. 10.1111/evo.13967 32246727

[mec16478-bib-0053] Moen, D. , & Morlon, H. (2014). Why does diversification slow down? Trends in Ecology and Evolution, 29(4), 190–197. 10.1016/j.tree.2014.01.010 24612774

[mec16478-bib-0054] Morlon, H. (2014). Phylogenetic approaches for studying diversification. Ecology Letters, 17(4), 508–525. 10.1111/ele.12251 24533923

[mec16478-bib-0055] Morlon, H. , Kemps, B. D. , Plotkin, J. B. , & Brisson, D. (2012). Explosive radiation of a bacterial species group. Evolution, 66(8), 2577–2586. 10.1111/j.1558-5646.2012.01598.x 22834754 PMC3871994

[mec16478-bib-0056] Morlon, H. , Lewitus, E. , Condamine, F. L. , Manceau, M. , Clavel, J. , & Drury, J. (2016). RPANDA: An R package for macroevolutionary analyses on phylogenetic trees. Methods in Ecology and Evolution, 7(5), 589–597. 10.1111/2041-210X.12526

[mec16478-bib-0057] Morlon, H. , Parsons, T. L. , & Plotkin, J. B. (2011). Reconciling molecular phylogenies with the fossil record. Proceedings of the National Academy of Sciences, 108(39), 16327–16332. 10.1073/pnas.1102543108 PMC3182748 21930899

[mec16478-bib-0058] Morton, J. B. (1990). Species and clones of arbuscular mycorrhizal fungi (Glomales, Zygomycetes): their role in macro‐ and microevolutionary processes. Mycotaxon (USA), 37, 493–515.

[mec16478-bib-0059] Munkvold, L. , Kjøller, R. , Vestberg, M. , Rosendahl, S. , & Jakobsen, I. (2004). High functional diversity within species of arbuscular mycorrhizal fungi. New Phytologist, 164(2), 357–364. 10.1111/j.1469-8137.2004.01169.x 33873553

[mec16478-bib-0060] Nathan, R. , Schurr, F. M. , Spiegel, O. , Steinitz, O. , Trakhtenbrot, A. , & Tsoar, A. (2008). Mechanisms of long‐distance seed dispersal. Trends in Ecology & Evolution, 23(11), 638–647. 10.1016/j.tree.2008.08.003 18823680

[mec16478-bib-0061] Öpik, M. , Davison, J. , Moora, M. , & Zobel, M. (2014). DNA‐based detection and identification of Glomeromycota: The virtual taxonomy of environmental sequences. Botany‐Botanique, 92(2), 135–147. 10.1139/cjb-2013-0110

[mec16478-bib-0062] Öpik, M. , Vanatoa, A. , Vanatoa, E. , Moora, M. , Davison, J. , Kalwij, J. M. , Reier, Ü. , & Zobel, M. (2010). The online database MaarjAM reveals global and ecosystemic distribution patterns in arbuscular mycorrhizal fungi (Glomeromycota). New Phytologist, 188(1), 223–241. 10.1111/j.1469-8137.2010.03334.x 20561207

[mec16478-bib-0063] Paradis, E. , Claude, J. , & Strimmer, K. (2004). APE: Analyses of phylogenetics and evolution in R language. Bioinformatics, 20(2), 289–290. 10.1093/bioinformatics/btg412 14734327

[mec16478-bib-0064] Pärtel, M. , Öpik, M. , Moora, M. , Tedersoo, L. , Szava‐Kovats, R. , Rosendahl, S. , Rillig, M. C. , Lekberg, Y. , Kreft, H. , Helgason, T. , Eriksson, O. , Davison, J. , Bello, F. , Caruso, T. , & Zobel, M. (2017). Historical biome distribution and recent human disturbance shape the diversity of arbuscular mycorrhizal fungi. New Phytologist, 216(1), 227–238. 10.1111/nph.14695 28722181

[mec16478-bib-0065] Perez‐Lamarque, B. , & Morlon, H. (2019). Characterizing symbiont inheritance during host–microbiota evolution: Application to the great apes gut microbiota. Molecular Ecology Resources, 19(6), 1659–1671. 10.1111/1755-0998.13063 31325911

[mec16478-bib-0066] Perez‐Lamarque, B. , Selosse, M. A. , Öpik, M. , Morlon, H. , & Martos, F. (2020). Cheating in arbuscular mycorrhizal mutualism: A network and phylogenetic analysis of mycoheterotrophy. New Phytologist, 226(6), 1822–1835. 10.1111/nph.16474 32022272

[mec16478-bib-0067] Pons, J. , Barraclough, T. G. , Gomez‐Zurita, J. , Cardoso, A. , Duran, D. P. , Hazell, S. , Kamoun, S. , Sumlin, W. D. , & Vogler, A. P. (2006). Sequence‐based species delimitation for the DNA taxonomy of undescribed insects. Systematic Biology, 55(4), 595–609. 10.1080/10635150600852011 16967577

[mec16478-bib-0068] Powell, J. R. , Monaghan, M. T. , Öpik, M. , & Rillig, M. C. (2011). Evolutionary criteria outperform operational approaches in producing ecologically relevant fungal species inventories. Molecular Ecology, 20(3), 655–666. 10.1111/j.1365-294X.2010.04964.x 21199026

[mec16478-bib-0069] Quince, C. , Curtis, T. P. , & Sloan, W. T. (2008). The rational exploration of microbial diversity. ISME Journal, 2(10), 997–1006. 10.1038/ismej.2008.69 18650928

[mec16478-bib-0070] R Core Team (2020). R: A language and environment for statistical computing. R Foundation for Statistical Computing.

[mec16478-bib-0071] Rabosky, D. L. (2009). Ecological limits and diversification rate: Alternative paradigms to explain the variation in species richness among clades and regions. Ecology Letters, 12(8), 735–743. 10.1111/j.1461-0248.2009.01333.x 19558515

[mec16478-bib-0072] Rabosky, D. L. (2016). Challenges in the estimation of extinction from molecular phylogenies: A response to Beaulieu and O’Meara. Evolution, 70(1), 218–228. 10.1111/evo.12820 26593734

[mec16478-bib-0073] Rabosky, D. L. , & Lovette, I. J. (2008). Density‐dependent diversification in North American wood warblers. Proceedings of the Royal Society B: Biological Sciences, 275(1649), 2363–2371. 10.1098/rspb.2008.0630 PMC2603228 18611849

[mec16478-bib-0074] Ramírez‐Barahona, S. , Sauquet, H. , & Magallón, S. (2020). The delayed and geographically heterogeneous diversification of flowering plant families. Nature Ecology and Evolution, 4(9), 1232–1238. 10.1038/s41559-020-1241-3 32632260

[mec16478-bib-0075] Read, D. J. (1991). Mycorrhizas in ecosystems. Experientia, 47(4), 376–391. 10.1007/BF01972080

[mec16478-bib-0076] Revell, L. J. (2012). Phytools: An R package for phylogenetic comparative biology (and other things). Methods in Ecology and Evolution, 3(2), 217–223. 10.1111/j.2041-210X.2011.00169.x

[mec16478-bib-0077] Rich, M. K. , Nouri, E. , Courty, P.‐E. , & Reinhardt, D. (2017). Diet of arbuscular mycorrhizal fungi: Bread and butter? Trends in Plant Science, 22(8), 652–660. 10.1016/j.tplants.2017.05.008 28622919

[mec16478-bib-0078] Rimington, W. R. , Pressel, S. , Duckett, J. G. , Field, K. J. , Read, D. J. , & Bidartondo, M. I. (2018). Ancient plants with ancient fungi: Liverworts associate with early‐diverging arbuscular mycorrhizal fungi. Proceedings of the Royal Society B: Biological Sciences, 285(1888), 20181600. 10.1098/rspb.2018.1600 PMC6191707 30305437

[mec16478-bib-0079] Rolland, J. , Condamine, F. L. , Jiguet, F. , & Morlon, H. (2014). Faster speciation and reduced extinction in the tropics contribute to the mammalian latitudinal diversity gradient. PLoS Biology, 12(1), e1001775. 10.1371/journal.pbio.1001775 24492316 PMC3904837

[mec16478-bib-0080] Royer, D. L. , Berner, R. A. , Montañez, I. P. , Tabor, N. J. , & Beerling, D. J. (2004). CO2 as a primary driver of Phanerozoic climate. GSA Today, 14(3), 4. 10.1130/1052-5173(2004)014<4:CAAPDO>2.0.CO;2

[mec16478-bib-0081] Säle, V. , Palenzuela, J. , Azcón‐Aguilar, C. , Sánchez‐Castro, I. , da Silva, G. A. , Seitz, B. , Sieverding, E. , van der Heijden, M. G. A. , & Oehl, F. (2021). Ancient lineages of arbuscular mycorrhizal fungi provide little plant benefit. Mycorrhiza, 31(5), 559–576, 10.1007/s00572-021-01042-5 34327560 PMC8484173

[mec16478-bib-0082] Sanders, I. R. (2003). Preference, specificity and cheating in the arbuscular mycorrhizal symbiosis. Trends in Plant Science, 8(4), 143–145. 10.1016/S1360-1385(03)00012-8 12711222

[mec16478-bib-0083] Sauquet, H. , & Magallón, S. (2018). Key questions and challenges in angiosperm macroevolution. New Phytologist, 219(4), 1170–1187. 10.1111/nph.15104 29577323

[mec16478-bib-0084] Savary, R. , Masclaux, F. G. , Wyss, T. , Droh, G. , Cruz Corella, J. , Machado, A. P. , Morton, J. B. , & Sanders, I. R. (2018). A population genomics approach shows widespread geographical distribution of cryptic genomic forms of the symbiotic fungus Rhizophagus irregularis. ISME Journal, 12(1), 17–30. 10.1038/ismej.2017.153 29027999 PMC5739010

[mec16478-bib-0085] Selosse, M.‐A. , & Le Tacon, F. (1998). The land flora: a phototroph‐fungus partnership? Trends in Ecology & Evolution, 13(1), 15–20. 10.1016/S0169-5347(97)01230-5 21238179

[mec16478-bib-0086] Sepp, S. K. , Davison, J. , Jairus, T. , Vasar, M. , Moora, M. , Zobel, M. , & Öpik, M. (2019). Non‐random association patterns in a plant–mycorrhizal fungal network reveal host–symbiont specificity. Molecular Ecology, 28(2), 365–378. 10.1111/mec.14924 30403423

[mec16478-bib-0087] Simon, L. , Bousquet, J. , Lévesque, R. C. , & Lalonde, M. (1993). Origin and diversification of endomycorrhizal fungi and coincidence with vascular land plants. Nature, 363(6424), 67–69. 10.1038/363067a0

[mec16478-bib-0088] Simon, L. , Lalonde, M. , & Bruns, T. D. (1992). Specific amplification of 18S fungal ribosomal genes from vesicular‐arbuscular endomycorrhizal fungi colonizing roots. Applied and Environmental Microbiology, 58(1), 291–295. 10.1128/aem.58.1.291-295.1992 1339260 PMC195206

[mec16478-bib-0089] Simpson, G. G. (1953). The major features of evolution. Columbia University Press.

[mec16478-bib-0090] Smith, S. E. , & Read, D. J. (2008). Mycorrhizal Symbiosis. Mycorrhizal Symbiosis. Elsevier. 10.1016/B978-0-12-370526-6.X5001-6

[mec16478-bib-0091] Stadler, T. (2011). Mammalian phylogeny reveals recent diversification rate shifts. Proceedings of the National Academy of Sciences, 108(15), 6187–6192. 10.1073/pnas.1016876108 PMC3076834 21444816

[mec16478-bib-0092] Strullu‐Derrien, C. , Selosse, M.‐A.‐A. , Kenrick, P. , & Martin, F. M. (2018). The origin and evolution of mycorrhizal symbioses: from palaeomycology to phylogenomics. New Phytologist, 220(4), 1012–1030. 10.1111/nph.15076 29573278

[mec16478-bib-0093] Stürmer, S. L. (2012). A history of the taxonomy and systematics of arbuscular mycorrhizal fungi belonging to the phylum Glomeromycota. Mycorrhiza, 22(4), 247–258. 10.1007/s00572-012-0432-4 22391803

[mec16478-bib-0094] Taberlet, P. , Bonin, A. , Zinger, L. , & Coissac, E. (Eds.), (2018). DNA amplification and multiplexing. In Environmental DNA. (pp. 41–57). Oxford Uni.

[mec16478-bib-0095] Tajima, F. (1983). Evolutionary relationship of DNA sequences in finite populations. Genetics, 105(2), 437–460. 10.1093/genetics/105.2.437 6628982 PMC1202167

[mec16478-bib-0096] Tedersoo, L. , Albertsen, M. , Anslan, S. , & Callahan, B. (2021). Perspectives and benefits of high‐throughput long‐read sequencing in microbial ecology. Applied and Environmental Microbiology, 87(17), 1–19. 10.1128/AEM.00626-21 PMC8357291 34132589

[mec16478-bib-0097] Tisserant, E. , Malbreil, M. , Kuo, A. , Kohler, A. , Symeonidi, A. , Balestrini, R. , Charron, P. , Duensing, N. , Frei dit Frey, N. , Gianinazzi‐Pearson, V. , Gilbert, L. B. , Handa, Y. , Herr, J. R. , Hijri, M. , Koul, R. , Kawaguchi, M. , Krajinski, F. , Lammers, P. J. , Masclaux, F. G. , … Martin, F. (2013). Genome of an arbuscular mycorrhizal fungus provides insight into the oldest plant symbiosis. Proceedings of the National Academy of Sciences of the United States of America, 110(50), 20117–20122. 10.1073/pnas.1313452110 24277808 PMC3864322

[mec16478-bib-0098] Toussaint, A. , Bueno, G. , Davison, J. , Moora, M. , Tedersoo, L. , Zobel, M. , Öpik, M. , & Pärtel, M. (2020). Asymmetric patterns of global diversity among plants and mycorrhizal fungi. Journal of Vegetation Science, 31(2), 355–366. 10.1111/jvs.12837

[mec16478-bib-0099] Upham, N. S. , Esselstyn, J. A. , & Jetz, W. (2019). Inferring the mammal tree: Species‐level sets of phylogenies for questions in ecology, evolution, and conservation. PLoS Biology, 17(12), e3000494. 10.1371/journal.pbio.3000494 31800571 PMC6892540

[mec16478-bib-0100] van der Heijden, M. G. A. A. , Martin, F. M. , Selosse, M.‐A.‐A. , & Sanders, I. R. (2015). Mycorrhizal ecology and evolution: the past, the present, and the future. New Phytologist, 205(4), 1406–1423. 10.1111/nph.13288 25639293

[mec16478-bib-0101] Varga, T. , Krizsán, K. , Földi, C. , Dima, B. , Sánchez‐García, M. , Sánchez‐Ramírez, S. , Szöllősi, G. J. , Szarkándi, J. G. , Papp, V. , Albert, L. , Andreopoulos, W. , Angelini, C. , Antonín, V. , Barry, K. W. , Bougher, N. L. , Buchanan, P. , Buyck, B. , Bense, V. , Catcheside, P. , … Nagy, L. G. (2019). Megaphylogeny resolves global patterns of mushroom evolution. Nature Ecology & Evolution, 3(4), 668–678. 10.1038/s41559-019-0834-1 30886374 PMC6443077

[mec16478-bib-0102] Venice, F. , Ghignone, S. , Salvioli di Fossalunga, A. , Amselem, J. , Novero, M. , Xianan, X. , & Bonfante, P. (2020). At the nexus of three kingdoms: the genome of the mycorrhizal fungus Gigaspora margarita provides insights into plant, endobacterial and fungal interactions. Environmental Microbiology, 22(1), 122–141. 10.1111/1462-2920.14827 31621176

[mec16478-bib-0103] Werner, G. D. A. , Cornelissen, J. H. C. , Cornwell, W. K. , Soudzilovskaia, N. A. , Kattge, J. , West, S. A. , & Kiers, E. T. (2018). Symbiont switching and alternative resource acquisition strategies drive mutualism breakdown. Proceedings of the National Academy of Sciences, 115(20), 5229–5234. 10.1073/pnas.1721629115 PMC5960305 29712857

[mec16478-bib-0104] Werner, G. D. A. , Cornwell, W. K. , Sprent, J. I. , Kattge, J. , & Kiers, E. T. (2014). A single evolutionary innovation drives the deep evolution of symbiotic N2‐fixation in angiosperms. Nature Communications, 5(1), 4087. 10.1038/ncomms5087 PMC4059933 24912610

[mec16478-bib-0105] Yildirir, G. , Malar, M. , Kokkoris, V. , & Corradi, N. (2020). Parasexual and sexual reproduction in arbuscular mycorrhizal fungi: Room for both. Trends in Microbiology, 28(7), 517–519. 10.1016/j.tim.2020.03.013 32360097

[mec16478-bib-0106] Zanne, A. E. , Tank, D. C. , Cornwell, W. K. , Eastman, J. M. , Smith, S. A. , FitzJohn, R. G. , McGlinn, D. J. , O’Meara, B. C. , Moles, A. T. , Reich, P. B. , Royer, D. L. , Soltis, D. E. , Stevens, P. F. , Westoby, M. , Wright, I. J. , Aarssen, L. , Bertin, R. I. , Calaminus, A. , Govaerts, R. , … Beaulieu, J. M. (2014). Three keys to the radiation of angiosperms into freezing environments. Nature, 506(7486), 89–92. 10.1038/nature12872 24362564

[mec16478-bib-0107] Ziegler, A. M. , Eshel, G. , McAllister Rees, P. , Rothfus, T. A. , Rowley, D. B. , & Sunderlin, D. (2003). Tracing the tropics across land and sea: Permian to present. Lethaia, 36(3), 227–254. 10.1080/00241160310004657

